# Part 2 – Coronary angiography with gadofosveset trisodium: a prospective intra-subject comparison for dose optimization for 100 % efficiency imaging

**DOI:** 10.1186/s12872-015-0152-8

**Published:** 2016-03-22

**Authors:** Mark A. Ahlman, Fabio S. Raman, Jianing Pang, Filip Zemrak, Veit Sandfort, Scott R. Penzak, Zhaoyang Fan, Songtao Liu, Debiao Li, David A. Bluemke

**Affiliations:** 1 National Institutes of Health, Radiology and Imaging Sciences, Clinical Center, 10 Center Drive, Building 10, Rm B1N264B.7, Bethesda, MD 20892 USA; 2 Medical Scientist Training Program, University of Alabama at Birmingham, Birmingham, AL USA; 3 Bioengineering, Cedars-Sinai Medical Center, Los Angeles, CA USA; 4 William Harvey Research Institute, Centre for Advanced Cardiovascular Imaging, Barts & The London School of Medicine & Dentistry, London, UK; 5 Department of Pharmacotherapy, University of North Texas, Fort Worth, Texas USA

**Keywords:** Gadofosveset trisodium, MS-325, Gadolinium-based intravascular contrast agent, Whole-heart coronary magnetic resonance angiography, Navigator-based angiography, 3.0 Tesla, Image quality, Respiratory motion correction

## Abstract

**Background:**

Three tesla (3T) coronary magnetic resonance angiography (MRA) may be optimized using gadolinium-based contrast agents (GBCA) such as gadofosveset trisodium. The goal of this study was to evaluate if there is a qualitative or quantitative improvement in the coronary arteries with variation in contrast dose.

**Methods:**

Twenty-eight healthy volunteers were prospectively recruited for coronary MRA at 3T using a steady state injection technique for 3D radial whole-heart image acquisition with retrospective respiratory self-gating (ClinicalTrials.gov identifier: NCT01853592). Nineteen volunteers completed both single- and double-dose imaging instances (0.03 and 0.06 mmol/kg, respectively). Intra-individual comparison of image quality was assessed by measurement of apparent signal/contrast-to-noise ratio (aSNR/aCNR) and subjective evaluation of image quality by 2 independent reviewers.

**Results:**

The average duration of coronary MRA acquisition was 7.2 ± 1.2 min. There was significantly higher (60 %, *p* < 0.001) aSNR of the aorta and right/left ventricle for the double dose compared to single dose injection scheme and aSNR of the coronary arteries increased by 70 % (*p* < 0.001) for the double dose injection. aCNR increased by +55 % and +60 % in the ventricles and coronary arteries, respectively (*p* < 0.001). Overall segmental artery visualization for single dose was possible 47 % of the time, which improved to 60 % with double dose (*p* = 0.019), predominantly driven by improvements in more distal segment visualization (+40 % improvement in mid arterial segments, *p* = 0.013).

**Conclusions:**

Gadofosveset trisodium dose of 0.06 mmol/kg significantly quantitatively and qualitatively improves the coronary artery image quality compared to 0.03 mmol/kg at 3T for moderate duration (6–8 min) steady state contrast enhanced coronary MRA.

## Background

Coronary artery disease (CAD) remains a leading cause of morbidity and mortality in the world. CT coronary angiography (CCTA) is the reference standard for non-invasive cross-sectional imaging of the coronary arteries. Coronary MRA can often be combined with venography, and comprehensive evaluation of myocardial function and structure. In addition, concerns regarding iodinated contrast allergy or concerns about ionizing radiation make coronary MRA appealing [1, 2]. However, coronary MRA remains technology challenging, especially at 3 Tesla compared to CCTA [3]. Therefore, further optimization is needed.

In general, coronary MRA has long scan times (5–15 min) because high resolution, wide spatial coverage and prospective respiratory gating via a diaphragmatic navigator has been required. Steady state free precession (SSFP) imaging is effective at defining coronary anatomy at 1.5T [4]. However at 3T, B0 and B1 field inhomogeneity and specific absorption rate (SAR) considerations result in limitations for a noncontrast approach. Contrast enhanced whole-heart imaging utilizing an inversion recovery technique is an alternative [5]. Long scan duration precludes the use of extracellular GBCAs, which have very short intravascular residence times (less than 1 min). An alternative approach uses an intravascular contrast agent such as gadofosveset trisodium (Ablavar®, Lantheus Medical Imaging, North Billerica, MA, USA). Gadofosveset has a high affinity for albumin, which resides predominately in the intravascular compartment, thus resulting in much longer enhancement of blood vessels compared to conventional extravascular MRI contrast agents (~10 min vs. 30 s, respectively) [6]. Gadofosveset has been validated at 1.5T for angiography of aortoiliac atherosclerotic disease using a dose of 0.03 mmol/kg [4, 7]. However, unlike the relatively motion-free aorta and iliac arteries, coronary MRA is complicated by the need to account for cardiac and coronary artery motion. Thus, the optimal dose of gadofosveset for coronary MRA at 3T had not been determined. For peripheral MRA, so-called “double-doses” of conventional GBCAs have shown to significantly increase raw signal intensity, SNR, and CNR [8, 9]. Double dosing is also commonly applied to cardiac MRA [10].

Advances in coronary whole-heart MRA acquisition with 100 % gating efficiency reduces acquisition time for coronary MRA by a factor of 2.5 to 3 or more [11, 12]. Thus, a coronary MRA duration of less than 10 min mimics our initial development of peripheral contrast enhanced MRA that was optimized for this timing with gadofosveset, intended for use in “double dose” gadolinium injection schemes [13].

The primary purpose of this study was to determine if the diagnostic quality and image characteristics of contrast-enhanced coronary magnetic resonance angiography (MRA) at 3T is improved by using a higher dose of gadofosveset combined with 100 % efficient whole heart coronary MRA. A secondary goal was to assess the patient tolerance and safety of the high versus low dose gadofosveset injection scheme.

## Methods

### Study population

Following approval by the National Heart, Lung, and Blood Institute institutional review board at the National Institutes of Health (NCT01853592), 28 subjects were recruited with written informed consent. Subjects were included between the ages of 18–45. Exclusion criteria contained standard MRI safety criteria, pregnancy, GBCA administration within 30 days, history of severe asthma, diagnosis of CAD, liver or kidney failure, and history of arrhythmia. After a focused history and physical exam, serum human chorionic gonadotropin and creatinine were drawn to exclude pregnancy or kidney failure, respectively. A complete blood count was drawn to screen for blood dyscrasia or other hematologic abnormality. Immediately prior to imaging, weight, height, heart rate, and blood pressure were recorded.

### MR angiography

Standard gadofosveset dosing followed the package insert for a single dose of 0.12 mL/kg (0.03 mmol/kg), whereas the double dose protocol used 0.24 mL/kg (0.06 mmol/kg). Using a previously validated dual-injection technique specific to gadofosveset [13], the dose was diluted to 50 mL with normal saline. A more rapid injection (1.5 mL/sec) phase was immediately followed by a continuous slow infusion (0.04 mL/sec), designed to maintain steady-state intravascular concentration. The rapid/slow infusion volume ratio was 60 %/40 %. All exams used a single Spectris Solaris EP (MEDRAD Inc., Pittsburgh, PA, USA) GBCA injector. Subjects underwent the double dose protocol, a 30-day waiting period, and the single dose protocol. If heart rate was greater than 70 beats per min prior to imaging, patients received up to 100 mg oral metoprolol tartrate (Mylan Pharmaceuticals, Canonsburg, Pennsylvania, USA).

MR data was collected using a 3T MAGNETOM Verio (Siemens Medical Solutions, Erlangen, Germany) with a 32 channel receiver coil array using a contrast-enhanced, free-breathing, electrocardiogram (ECG)-gated, fat-saturated and inversion-recovery prepared spoiled gradient echo sequence with the following parameters: TE/TR = 3.5/1.8 ms, inversion time = 350 ms, bandwidth = 704 Hz/pixel, field of view = 400^3^ mm^3^, matrix size = 384^3^, 3D radial k-space trajectory, number of projections = 12,000. The trigger delay and acquisition window length in ECG gating were set according to the mid-diastolic quiescent period. No prospective respiratory gating was performed. Rather, respiratory motion-corrected reconstruction was performed retrospectively offline using a previously validated method, whereby the raw MR data was binned into respiratory phases based on the self-navigation projections, and the respiratory motion was corrected using an image-based registration approach [11, 12].

Immediately following imaging, the subjects were asked open-ended questioning regarding any uncomfortable side effects due to contrast infusion, which were subjectively graded by the participant on a scale of 0 (none) to 10 (severe).

### Image processing and analysis

Using Osirix™ version 5.8.5 (Pixmeo SARL, Geneva, Switzerland), the aSNR and aCNR were calculated in reference to the myocardium by drawing large regions of interest (ROI) in the left ventricle (242 ± 64 mm^2^), right ventricle (184 ± 62 mm^2^), descending aorta (75 ± 22 mm^2^), and ascending aorta (163 ± 65 mm^2^). Likewise, a smaller ROI was drawn for the coronary left main (LM) artery in the same plane that it branched from the ascending aorta (6 ± 2 mm^2^). However, as the spatial resolution limited accurate ROIs being drawn for coronary arterial lumen, aSNR and aCNR values were obtained by averaging individual pixel densities of three pixels within the proximal arterial lumen (pixel size = 1.04 mm^2^) for the right coronary artery (RCA), left anterior descending (LAD), and left circumflex (LCX) coronary arteries. For the reference myocardium, three separate ROIs were drawn over the septum (each 12 ± 1.4mm^2^) in the two-chamber view and signal intensities were averaged. Similarly, background air intensity was calculated using a large ROI superior to the chest cavity (25.5 ± 11.4 cm^2^). The equations used to calculate aSNR and aCNR (relative to myocardium) were then used as follows (SI – Signal Intensity, SD – Standard Deviation):

$$ aSNR=\frac{S{I}_{vessel}}{S{D}_{air}} $$

$$ aCNR=\frac{S{I}_{vessel}-S{I}_{myocardium}}{S{D}_{air}} $$

Two cardiologists experienced in coronary imaging (FZ, VS) performed blinded independent review of the coronary arteries for diagnostic quality in the LAD, LCX, and RCA in the proximal and mid portions of the artery. To measure overall difference in quality between dosing attempts, image quality was reported using a qualitative scale of 0 (artery segment not visualized), and 1 (visualized). For measurements of inter- and intra-reader agreement, a more gradated categorical scale was used: 0, not interpretable; 1, poor (severe artifacts); 2, good (mild to moderate artifacts); 3, very good (minimum to mild artifacts); 4, excellent (minimum or no artifacts).

Using a higher (0.05 mmol/kg) dose of Gadofosveset, prior studies have observed an approximately 50 % increase in CNR and 40 % increase in SNR in the peripheral and central vasculature compared to the standard 0.03 mmol/kg dosing scheme [14]. In a similar fashion, we separately evaluated aCNR and aSNR for gadofosveset coronary angiography [15]. Using this a priori information, we determined sample size for a paired comparison with beta = 0.8 and alpha = 0.05 to detect a 50 % increase in aCNR, and 40 % increase in aSNR for 0.06 mmol/kg compared to 0.03 mmol/kg gadofosveset. 19 subjects provided at least 80 % power; additional subjects were recruited to account for potential subject withdrawal prior to completion of two MRI scans.

Significant differences in quantitative and qualitative image quality parameters were calculated using Wilcoxon matched-pairs signed rank test. The intra- and inter-class correlation coefficient (ICC) was calculated for per-segment qualitative scores for single readers and between readers. Chi square analysis was performed for the determination if there was a higher proportion and severity of side effects due to double dosing among subjects who completed both single and double dose instances. Statistical significance was considered to have a *p*-value < 0.05. Differences in demographic, physiologic, and lab parameters between single dose and double dose scanning were compared with the Mann–Whitney or Chi square test, where appropriate.

## Results

### Study population

With demographics shown in Table 1, 22 of 28 subjects recruited were able to complete the initial double-dose imaging, of which, 19 completed the follow up single-dose imaging. A total of 9 subjects met exclusion criteria during the protocol because of: pre-screening lab values out of the normal range (4 subjects), lack of adequate intravenous access (1 subject), self-withdrawal prior to imaging (1 subject), or loss to follow-up following the initial MRI (3 subjects).

**Table 1:** Subject demographics

	Single dose (*n* = 19)	Double dose (*n* = 22)	*P* value
Age	29.6 ± 6.8	29.1 ± 6.7	NS
Male	26.3 % (5/19)	22.7 % (5/22)	NS
Height (cm)	165.4 ± 7.5	165.9 ± 7.3	NS
Weight (kg)	71.6 ± 20.2	70.9 ± 19.2	NS
Body mass index (kg/m^2^)	26.0 ± 6.2	25.6 ± 5.9	NS
Hematocrit (%)	41.1 ± 5.6	40.7 ± 6.1	NS
Creatine (mg/dL)	0.81 ± 0.16	0.80 ± 0.15	NS
eGFR (mL/min/1.73 m^2^)	113.0 ± 11.6	107.8 ± 12.1	NS
Heart rate (bpm)	66.7 ± 7.0	66.1 ± 11.0	NS
Systolic blood pressure (mmHg)	117.6 ± 11.5	117.1 ± 12.1	NS
Diastolic blood pressure (mmHg)	72.3 ± 9.9	70.1 ± 10.6	NS
Imaging Parameters			
Dose (mL)	8.6 ± 2.4	17.1 ± 4.7	**<**0.001
Imaging time (min)	6.8 ± 1.2	7.5 ± 1.2	NS

*NS* Non significant

MRA imaging time for double and single dose instances was not significantly different, with an overall average of 7.2 ± 1.2 min.

### Quantitative evaluation

Shown in Table 2, there were uniform, statistically significant increases in aSNR for all regions measured. aCNR also increased for all segments with the exception of the LCX (*p* = 0.09). Overall, there was an approximate 60 % increase in aSNR of the aorta and ventricles (*p* < 0.001) and an approximate 70 % increase (*p* < 0.001) in the coronary arteries. There was a similar increase in aCNR of the aorta/ventricles and coronary arteries, representing approximately +55 % (*p* < 0.001) and +60 % (*p* < 0.001), respectively.

**Table 2:** Quantitative aSNR and aCNR evaluation

	aSNR			aCNR		
Level	Single dose	Double dose	*P* value	Single dose	Double dose	*P* value
Left Ventricle	11.0 ± 4.5	17.3 ± 7.8	<0.001	6.0 ± 2.7	9.0 ± 5.0	0.002
Right Ventricle	10.7 ± 8.4	16.7 ± 11.7	<0.001	5.7 ± 6.8	8.4 ± 9.2	0.006
Desc. Aorta	9.5 ± 4.0	15.6 ± 8.1	<0.001	4.5 ± 2.9	7.2 ± 5.5	0.01
Prox. Aorta	13.1 ± 6.1	21.9 ± 10.6	<0.001	8.1 ± 4.5	13.6 ± 7.7	<0.001
Overall	11.1 ± 6.0	17.9 ± 9.8	<0.001	6.1 ± 4.6	9.5 ± 7.3	<0.001
LMS	10.5 ± 4.8	18.2 ± 8.1	<0.001	5.4 ± 3.6	9.6 ± 6.4	0.002
LAD	14.5 ± 4.3	23.6 ± 8.7	<0.001	9.0 ± 3.7	14.1 ± 8.3	0.02
LCX	10.7 ± 3.5	16.5 ± 4.3	<0.001	5.5 ± 3.1	7.8 ± 4.5	0.09
RCA	15.5 ± 6.6	25.3 ± 11.0	<0.001	10.4 ± 5.1	16.7 ± 10.0	0.01
Overall	12.8 ± 5.4	21.0 ± 9.1	<0.001	7.6 ± 4.5	12.1 ± 8.3	<0.001
Other						
Myocardium	4.9 ± 2.3	8.3 ± 3.8	<0.001			

There is higher aSNR and aCNR with double dose gadofosveset

*aSNR* Apparent signal-to-noise ratio

*aCNR* Apparent Contrast-to-noise ratio (relative to the myocardium)

*LM* Left main coronary artery

*LAD* Left anterior descending coronary artery

*LCX* Left circumflex coronary artery

*RCA* Right coronary artery

### Qualitative evaluation

Shown in Table 3, there were statistically significant improvements in qualitative vessel measurements with double dosing. Improvement was evidenced primarily in the mid portions of the artery (+40.6 %, *p* = 0.013). Although there was a uniform trend for an increase in vessel identification for all segments measured, statistically significant changes were not shown for the proximal LCX and proximal and distal RCA. With all proximal and mid segments included for evaluation, the overall vessel identification at single dose was 0.47 ± 0.27, which improved to 0.60 ± 0.25. The intra-and inter-class correlation coefficients were high for proximal coronary artery segments (ICC >0.7), with mixed results in the mid to distal segments.

**Table 3:** Qualitative evaluation

	Mean image quality			Wilcoxon	ICC	
Level	Single dose	Double dose	Δ %	*P* value	Intra	Inter
**LAD**_**prox**_	0.65 ± 0.40	0.89 ± 0.26	**36.9**	**0.004**	0.77	0.81
**LAD**_**mid**_	0.48 ± 0.33	0.60 ± 0.29	**25.0**	**0.048**	0.44	0.7
**LCX**_**prox**_	0.52 ± 0.39	0.62 ± 0.40	19.2	NS	0.73	0.76
**LCX**_**mid**_	0.14 ± 0.25	0.33 ± 0.35	**135.7**	**0.020**	0.6	0.51
**RCA**_**prox**_	0.68 ± 0.38	0.69 ± 0.40	1.5	NS	0.87	0.79
**RCA**_**mid**_	0.33 ± 0.24	0.43 ± 0.38	30.3	NS	0.48	0.61
**Overall**_**prox**_	0.62 ± 0.36	0.73 ± 0.29	17.7	NS	-	-
**Overall**_**mid**_	0.32 ± 0.22	0.45 ± 0.27	**40.6**	**0.013**	-	-
**Overall**_**prox+mid**_	0.47 ± 0.29	0.60 ± 0.25	**27.7**	**0.019**	-	-

Double dosing results in higher per-segment image quality of arterial segments. Statistically significant changes are shown in bold

*LAD* Left anterior descending coronary artery

*LCX* Left circumflex coronary artery

*RCA* Right coronary artery

*prox* Proximal

*mid* Middle

**Fig. 1:**
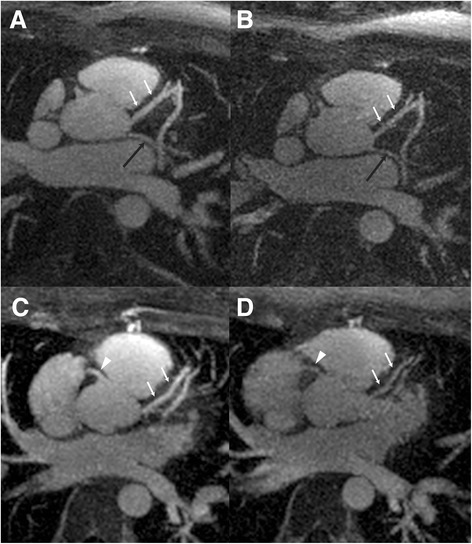
Visual comparison of image quality with double dose. Double dose (left column) and single dose (right column) maximum intensity projection MRA images of the heart for two subjects (top and bottom rows). Visually compared to single dose images (**b** and **d**), double dose images (**a** and **c**) show appreciably higher image quality of the LAD (white arrow), RCA (white arrowhead), and LCX arteries (black arrow). Window and level is fixed for representative image comparisons per subject

**Fig. 2:**
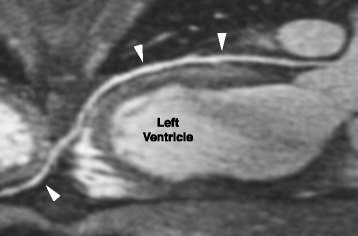
Curved multiplanar reconstruction of the LAD (white arrowheads) using an image acquired with the double dose steady state injection protocol. The LAD can be followed from the proximal (right) to the distal segment (left), and is seen wrapping around the apex of the left ventricle (far left)

**Fig. 3:**
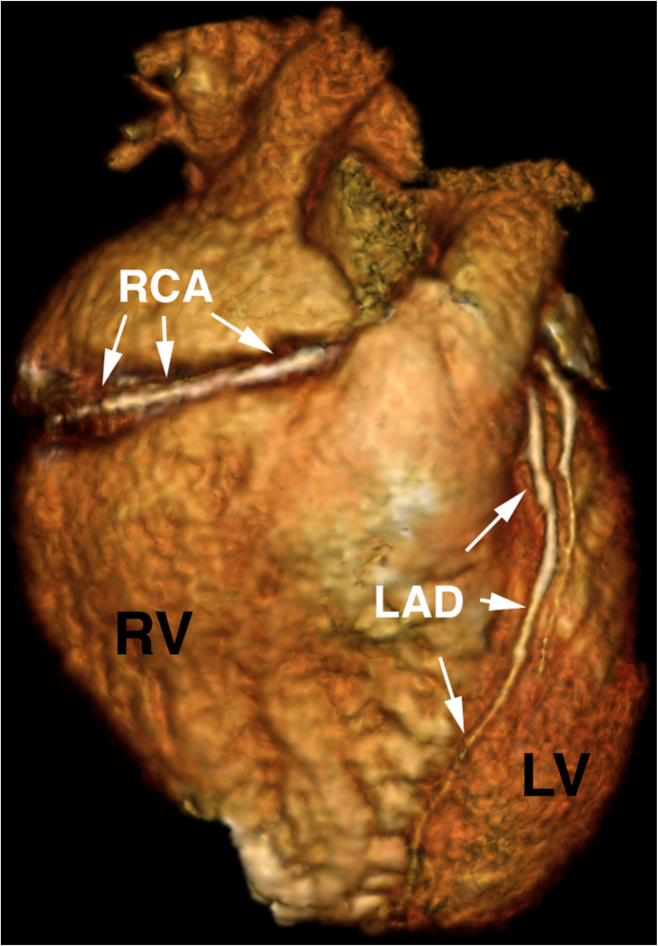
Example of a 3-dimensional projection of a coronary angiogram using a double dose injection protocol. The LAD can be followed to its most distal segment, wrapping around the apex of the heart

Images representative of a perceptible visual difference in image quality between the single and double dosing protocol is shown in Fig. 1. Curved multiplanar reconstruction and 3-dimensional volume surface reconstruction of the heart using the double dose protocol are shown in Figs. 2 and 3, respectively.

### Side effect severity

Following the initial double dose injection, one subject was excluded from further study because of the feeling of self-limiting “throat tightening” and throat pruritus. A second subject did not return for single dose scanning for undisclosed reasons. Of patients completing both examinations, more patients reported side effects of at least 1 out of 10 severity for double dose compared to single dose injection (65 % vs 42 %, *p* < 0.0001). Symptom severity was also higher (*p* < 0.0001) in double dose compared to single dose injection with a mean of 2.83 ± 2.61 and 1.13 ± 1.80, respectively. Ranked in order of occurrence, the most common reported side effects are shown in Table 4.

**Table 4:** Side effect profile

Times reported	Event	Attribution to gadofosveset or saline injection	Expected
12	Pelvic tingling	Probable	Yes
9	Pelvic itching	Probable	Yes
6	Arm chill/coldness/numbness/tingling	Probable	Yes
5	nausea	Probable	Yes
3	Nasal congestion	Unlikely	No
2	Metallic taste	Probable	Yes
2	Saline smell	Probable	Yes
2	Increased salivation	Probable	Yes
2	Warm sody sensation	Probable	Yes
1	Pelvic pressure	Probable	Yes
1	Pelvic discomfort	Probable	Yes
1	Low back pain	Unlikely	No
1	Prickly sensation	Probable	Yes
1	Cold body sensation	Probable	Yes
1	Tingling body sensation	Probable	Yes
1	Reflux sensation	Unlikely	No
1	Throat itching	Probable	Yes
1	Throat irritation	Probable	Yes

More than one symptom could be reported by a single participant

## Discussion

At 3.0-Tesla field strength, contrast-enhancement of the coronary arteries has been explored for MRA [5] and gadofosveset has shown promise in this application [16]. Previous use of gadofosveset in coronary MRA has shown a small increase in image quality compared to the more conventional MR contrast agents [15]. Furthermore, the long intravascular residence time and high T1 relaxivity may afford improvements in self-navigated respiratory motion compensation [17]. This strategy with gadofosveset is desirable for the 3D radial protocol used in this study as each k-space line contributes equally to the image contrast; therefore a stable contrast enhancement throughout the scan duration is desired. Additional diagnostic improvements with higher doses of gadofosveset have not been systematically evaluated with these advanced reconstruction techniques. Our results found higher aSNR and aCNR, as well as superior image quality represented by higher qualitative yield, particularly in the mid arterial segments. Our finding of significant increases in the more distal segments may be expected because of the opportunity to increase the otherwise poor luminal signal intensity at these levels because of small vessel caliber, compared to the proximal segments where single dose may suffice for visualization of the larger caliber artery at this level. Nonetheless, our results did show a statistically significant qualitative improvement in the proximal LAD. Despite a uniform trend for improved aCNR, aSNR, and qualitative measurement with double dose across all vascular territories, statistical significance was lacking in qualitative measurement in the LCx and RCA territory and with aCNR of the LCX, which may be due to limitations in field homogeneity, higher regional respiratory or cardiac motion, or limitations in sample size. With the intent for a robust evaluation of the entire coronary arterial tree with MR, there is promise in the combination of a higher dosing of gadofosveset along with other improvements in image acquisition [18].

Regarding the technique of coronary MRA with gadofosveset, we were able to target the image acquisition for the mid-diastolic quiescent period prior to contrast injection. Anecdotally, we observed an increase in the heart rate just after injection (5–15 beats per minute increase), which may have altered the mid-diastolic rest phase timing relative to the pre-planning cine images. In this regard, further improvements in image quality may follow with real-time adaptation to heart rate changes or other methods to mitigate the effect of heart rate variability [19], in addition to stabilization of heart rate with beta blockade [20].

Double dose injection protocols appear to predominate in current practice for cardiac MRI [10]. Increased risk of nephrogenic systemic sclerosis (NSF) as well as patient comfort are factors to consider for dose increases of gadolinium-based contrast agents [21]. We did not evaluate the long term safety of our dosing scheme, although gadofosveset safety of 0.03, 0.05, and 0.07 mmol/kg dosing has been previously reported [7]. Although patients reported an increase in the side effects of the higher dose of gadofosveset in this study, we did not observe severe contrast reactions for either dosing scheme. Because any abnormal liver or kidney function measurement resulted in exclusion from participation, the safety of higher doses of gadofosveset for patients with health complications is not evaluated here.

## Conclusions

The use of double doses of gadofosveset trisodium results in higher qualitative and quantitative image quality compared to the standard single dose. In combination with further improvements in technique, double dosing shows promise for use in coronary MR angiography at 3.0 Tesla. Diagnostic efficacy could be further evaluated to determine whether the superior image quality allows better assessment of coronary structural abnormalities in atherosclerotic patients.

## Abbreviations

3T: 3.0 Tesla
aCNR: apparent contrast-to-noise ratio
aSNR: apparent signal-to-noise ratio
CAD: coronary artery disease
CCTA: computed tomography coronary angiography
ECG: electrocardiogram
GBCA: gadolinium-based contrast agent
LAD: left anterior descending
LCX: left circumflex
LM: left main
Mid: middle
MR: magnetic resonance
MRA: magnetic resonance angiography
Myo: myocardium
NS: non significant
Prox: proximal
RCA: right coronary artery
ROI: region of interest
SD: standard deviation
SI: signal intensity
